# Supplementary material for: Cost-Efficient Expression of Human Cardiac Myosin Heavy Chain in C2C12 Cells with a Non-Viral Transfection Reagent
Source: Int J Mol Sci. 2024 Jun 19;25(12):6747. doi: 10.3390/ijms25126747 (PMC11203843; doi:10.3390/ijms25126747)
Supplement: Supplementary file 1 [file ijms-25-06747-s001.zip › Berg et al Supplemental information - updated in proof.pdf]

Cost-efficient expression of human cardiac myosin heavy chain in C2C12 cells with a non-viral transfection reagent

Albin E. Berg#, Lok Priya Velayuthan#, Alf Månsson*, Marko Usaj*

Department of Chemistry and BiomedicalSciences, Faculty of Health and Life Science, Linnaeus University, Kalmar, Sweden

*Corresponding authors
alf.mansson@lnu.se
marko.usaj@lnu.se

# Contributed equally

Supplemental information

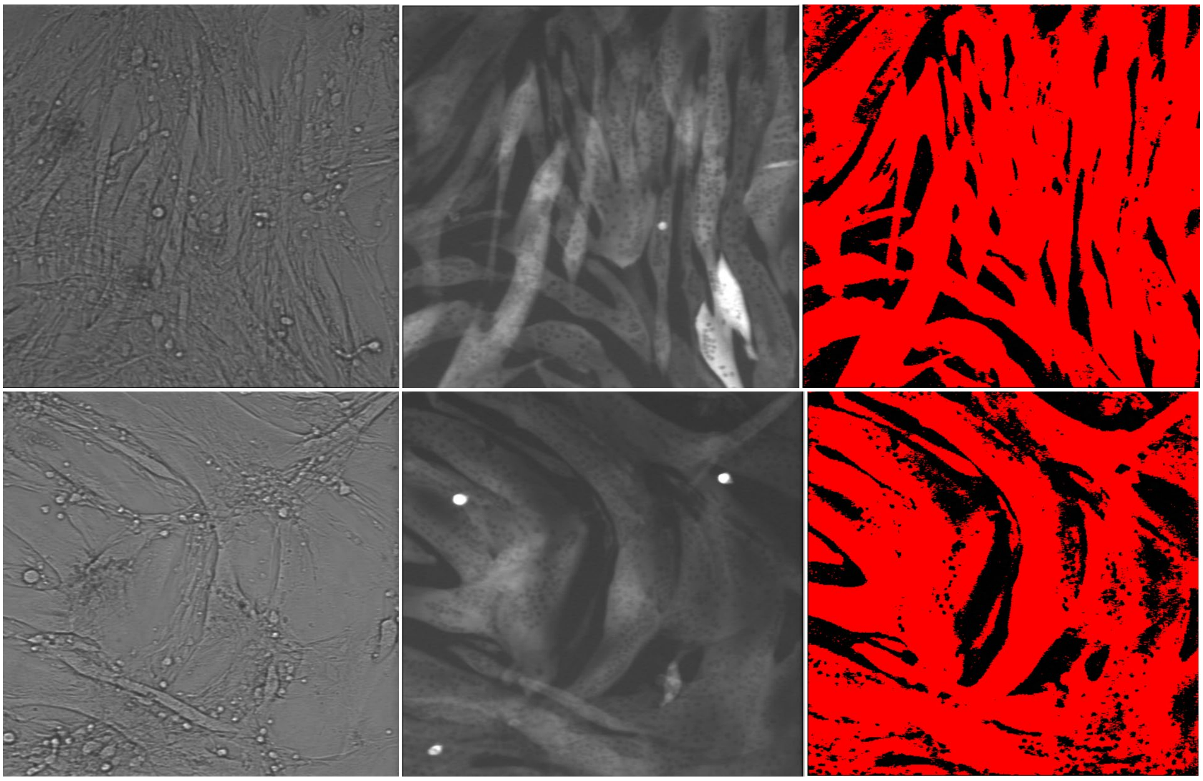

Figure S1: Two representative examples of image analysis in Fiji (script below) for quantifying transfection efficiency (see also Material and Methods in the main document). *Left:* brightfield microscopy images. Note that the cells (myotubes) are covering the entire field of view. *Middle:* eGFP fluorescence microscopy images of the same region show that (some of) the multinucleated myotubes are efficiently expressing the S1L-eGFP-FLAG myosin construct. *Right:* Segmentation results of the fluorescence images represent automatically detected transfected cell area (in red) expressing S1L-eGFP-FLAG which was then used to extract the mean grayscale level used for calculating transfection efficiency.

```
//To run on an open fluorescence image (get transfection area and intensity)
run("Subtract Background...", "rolling=50");

setAutoThreshold("Default dark");
//run("Threshold...");
setAutoThreshold("Default dark no-reset");
setThreshold(3, 255);//to be adapted based on exposure time, gain, etc.

run("Measure");
```

The script in Fiji used to determine the transfected area (%) and the expression intensity (the mean grayscale level) to calculate transfection efficiency.

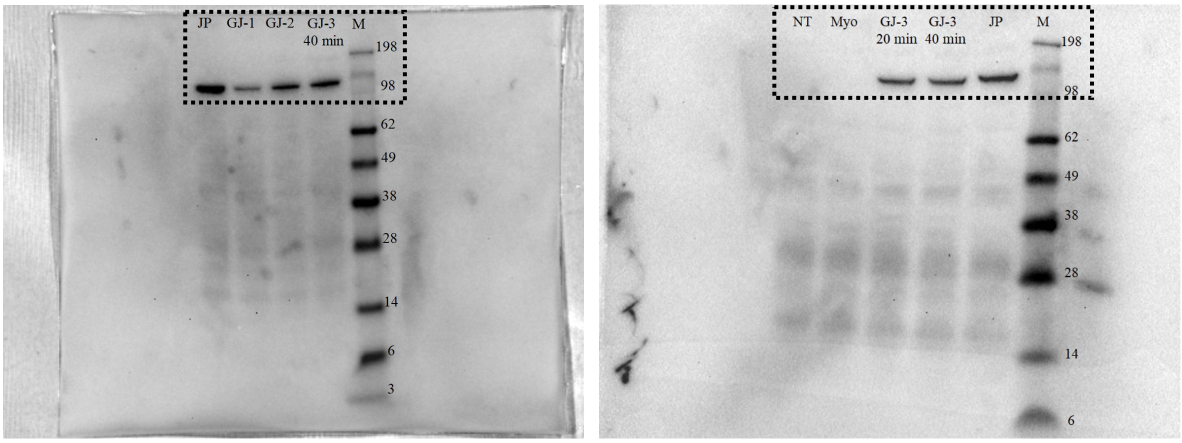

Figure S2. The original membrane of Western Blots using anti-FLAG antibodies for quantification of an expressed construct in total cell lysates at different transfection conditions (see Figure 2 in the main paper for details). The dashed rectangle represents the cropped area as presented in Figure 2 (main paper).

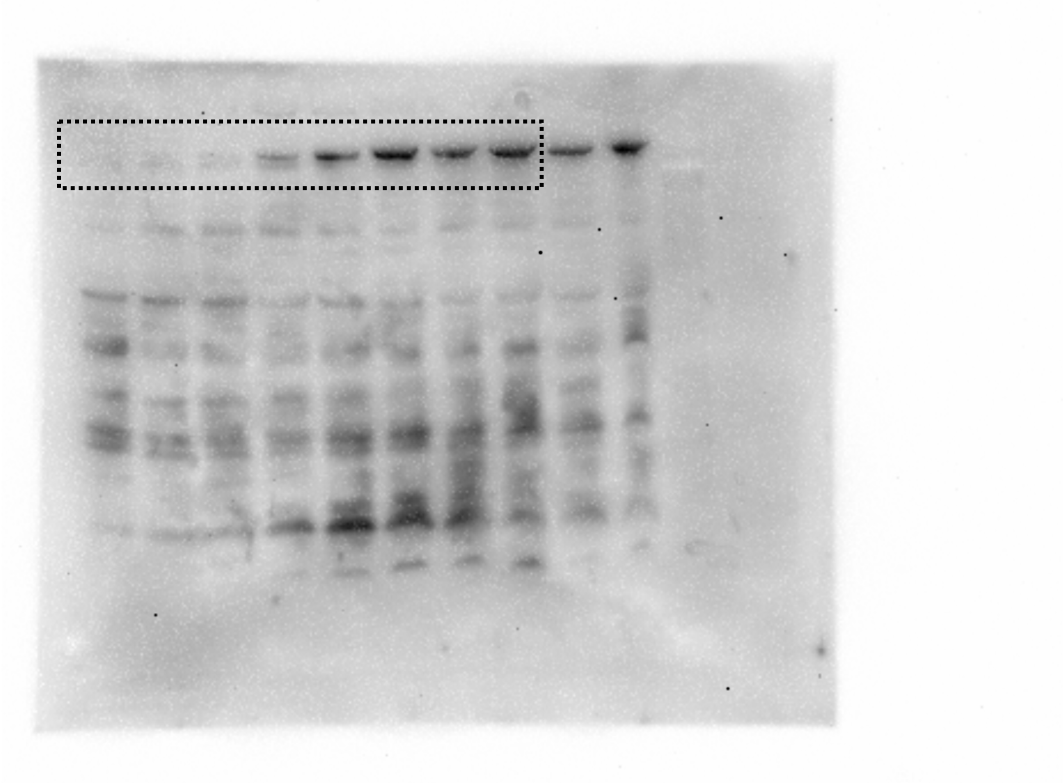

Figure S3. The original membrane of Western Blot using anti-FLAG antibodies for quantification of an expressed construct over time (1-8 days post-transfection). The dashed rectangle represents the cropped area as presented in Figure 3 (the main paper). The far-right samples are not related to the study.

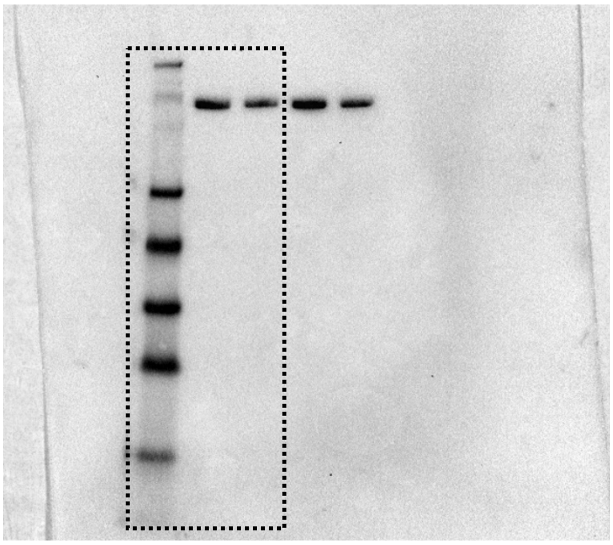

Figure S4. The original membrane of Western Blot using anti-FLAG antibodies for quantification of the expressed construct in eluate after protein purification. The dashed rectangle represents the cropped area as presented in Figure 4 (the main paper). The far-right samples are not related to this study.

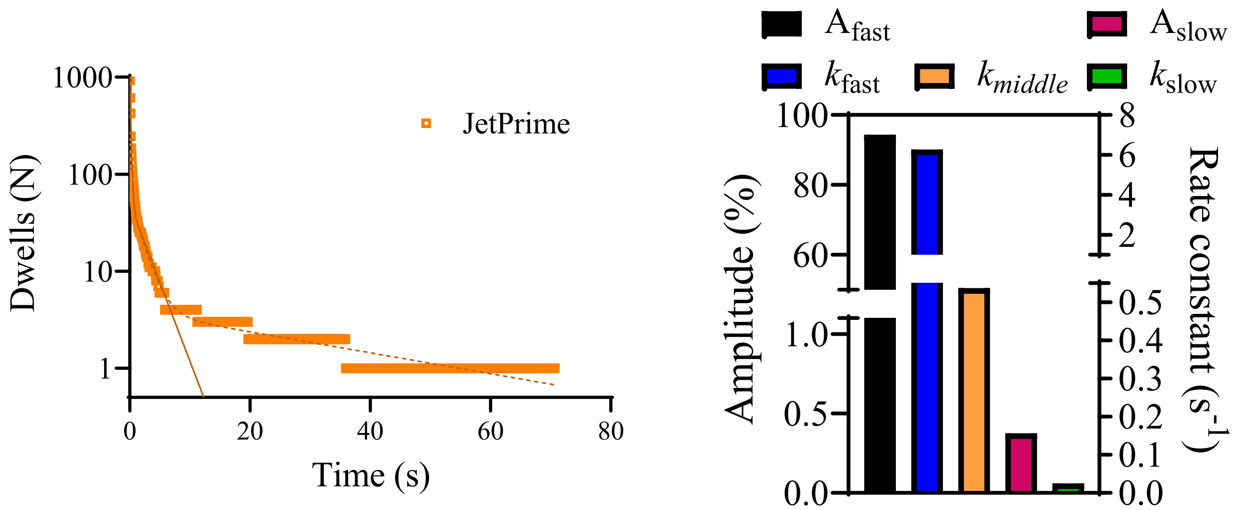

Figure S5. Alternative fit to JetPrime data from Figure 5G. *Left:* Cumulative frequency distributions of Alexa-nucleotide dwell time events from actin-activated ATPase activity from myosin construct purified from cells transfected with JetPrime. The solid line represents the actomyosin data fitted with double exponential functions as in the main document while the dashed line represents an alternative fit to triple exponential functions possible due to two longer dwells of 35.88 s and 70.668 s. Note that such long dwell times occasionally appear in single-molecule actomyosin experiments and we have attributed them[1] to myosin motors exhibiting basal ATP turnover either being accidentally surface-adsorbed outside the actin filaments or, for some reason, cross-linked to actin in a configuration that is not accessible for productive cycling with actin binding. *Right:* Amplitudes and rate constants from the fitting of data on the left, where the fast rate constant was attributed to actin-activated myosin ATPase activity (k_{cat}) while the slow rate constant was attributed to basal myosin ATPase activity (k_{basal}). The middle phase here represents the unspecific Alexa647-ATP binding to myosin molecules ($k_{unspecific}$). Error estimates refer to 95% confidence intervals derived in the regression analysis. Note that the fast and middle phases are very similar in amplitudes and rates from those obtained using double exponential fit (Figure 5H in the main paper). On the other hand, the slow phase is as expected very minor, represented by less than 0.5 % in amplitude. Due to that we were motivated to use the double exponential model in the main paper to facilitate comparison with GenJet data which lacked long dwells and could thus be fitted only by double exponential fit.

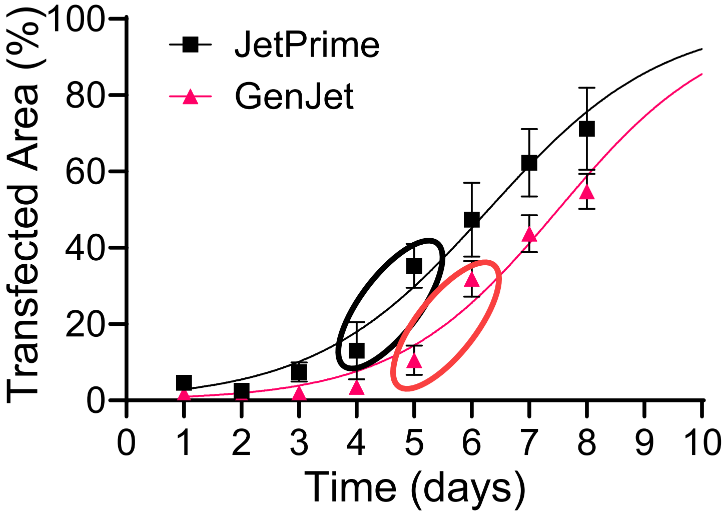

Figure S6. Transfected area expansion at each of 8 days post-transfection**.** The quantification of the transfected area expansion over time as a fraction of the area with eGFP fluorescence. GenJet data as in Figure 3 (main text) JetPrime data reproduced from [2]. Note the characteristic jump (encircled in black and red) in the transfection area to near identical values for both transfection reagents except that the jump is delayed for one day in case of GenJet transfection. Fitting the data to the logistic growth curve with its extrapolation to day 10 (to guide the eye) suggests that between day 8 and 9 the GenJet transfected area would reach that of JetPrime at day 7 (the standard cell harvesting day) post-transfection.

Supporting Movie legends

Movie S1.

Representative in vitro motility assay using purified myosin from cells transfected with GenJet after double "deadheading". Bar represents 10 µm.

Movie S2.

Fully differentiated C2C12 myotubes show a beating pattern after external electric stimulation. After 19 days in differentiation medium, C2C12 myoblasts show prominent myogenic differentiation into myotubes. By using platinum electrodes and an electric pulse generator (Grass S44 Stimulator), a train of electric pulses (5 V/cm, 1Hz, 2 ms) was delivered resulting in a beating pattern of myotubes. The delivery of electric pulses was controlled by observation using an oscilloscope (DSO6014A, Agilent Technologies).

Supporting References

1. Berg, A.; Velayuthan, L. P.; Tagerud, S.; Usaj, M.; Mansson, A., Probing actin-activated ATP turnover kinetics of human cardiac myosin II by single molecule fluorescence. *Cytoskeleton (Hoboken)* **2024**.
2. Velayuthan, L. P.; Moretto, L.; Tagerud, S.; Usaj, M.; Mansson, A., Virus-free transfection, transient expression, and purification of human cardiac myosin in mammalian muscle cells for biochemical and biophysical assays. *Scientific reports* **2023,** 13, (1), 4101.
